# Novel snake *Circovirus* from alpine pit viper (*Gloydius strauchi*) in China: evidence of a mammal-avian genetic recombinant

**DOI:** 10.3389/fmicb.2025.1725961

**Published:** 2026-01-08

**Authors:** Zhige Tian, Sirong Luo, Jiayi Li, Xingyu Liu, Yingxi Huang, Yuping Fan, Chenlei Zhou, Peng Guo, Xiaoliang Hu

**Affiliations:** Faculty of Agriculture, Forestry and Food Engineering, Yibin University, Yibin, China

**Keywords:** *Circovirus*, ecology, *Gloydius strauchi*, recombination, reptiles

## Abstract

**Introduction:**

Circoviruses within the family *Circoviridae* have been identified across diverse vertebrate taxa, including mammals, birds, and reptiles.

**Methods:**

This study investigated the oral cavity of the Asian pit viper (*Gloydius strauchi*) in southwestern China using PCR assay. The presence of *Circovirus* strain GsCV1 in oral samples was confirmed using PCR with consensus primers.

**Results and discussion:**

In this study, a recombinant *Circovirus* strain (GsCV1) was detected in the oral cavity of the Asian pit viper (*Gloydius strauchi*), a high-altitude species endemic to the plateaus (1,500–4,500 m) of Sichuan Province, China. Complete genome sequencing revealed a 1,811-bp circular DNA genome encoding two principal open reading frames for the replication-associated (Rep) and capsid (Cap) proteins, along with a conserved 9-bp nucleotide nonamer motif located at the apex of the stem-loop structure. Phylogenetic analysis indicated that the Rep sequence of GsCV1 clustered with mammalian circoviruses, whereas the Cap sequence was more closely related to avian strains. Recombination analysis suggested that GsCV1 emerged from interspecies recombination events involving bat-associated circovirus 3 (BatACV3), swan circovirus (SwCV), and zebra finch circovirus (ZfiCV). These findings expand the known host range and evolutionary complexity of circoviruses and raise the possibility that ecological behaviors and habitat-specific pressures in snakes may influence circoviral diversification. Further investigation is required to elucidate the prevalence, pathogenic potential, and ecological significance of circoviruses in reptilian hosts.

## 1 Introduction

Members of the family *Circoviridae*, comprising the genera *Circovirus* and *Cyclovirus* (Rosario et al., 2017), possess the smallest known non-enveloped single-stranded DNA (ssDNA) genomes among viruses, typically ranging from 1.7 to 2.1 kb in length. These compact circular genomes encode two major proteins, including the replication-associated (Rep) and capsid (Cap) proteins (Rosario et al., 2017). Circoviruses have been identified in over 76 vertebrate and invertebrate host species, including pigs (Allan et al., 2012), birds (Todd, 2000), canines (Anderson et al., 2017), minks (Lian et al., 2014), fish, bats, humans, and other mammals (Breitbart et al., 2017). To date, viruses from more than 11 families have been reported in reptiles, including *Iridoviridae* (Grosset et al., 2014; Papp and Marschang, 2019; Stöhr et al., 2013; Tamukai et al., 2016), *Adenoviridae* (Benge et al., 2019; Kim et al., 2002; Liu et al., 2023), *Parvoviridae* (Pénzes et al., 2015), *Paramyxoviridae* (Abbas et al., 2012), and *Papillomaviridae* (Herbst et al., 2009; Liu et al., 2023). Circoviruses have been detected in various reptilian hosts, such as turtles (black-breasted leaf turtle, Hermann’s tortoise, and common box turtle) (Tóth et al., 2024), snakes (black-headed python, speckled rattlesnake) (Altan et al., 2019; Gilbert et al., 2014), and lizards (e.g., *Pogona vitticeps*) (Chang et al., 2020). Our research team previously reported the presence of *Circovirus* in the oral cavity of *Gloydius angusticeps* from Sichuan province (Liu et al., 2023), raising the possibility of long-term adaptation and coevolution of circoviruses within ophidian hosts.

Circoviruses are recognized for their immunosuppressive effects, often predisposing infected hosts to secondary infections and compromising lymphoid organ integrity (Mankertz et al., 2004). For example, porcine circovirus 2 (PCV2) has been implicated in multiple disease syndromes in pigs, including post-weaning multisystemic wasting syndrome (PMWS), porcine dermatitis and nephropathy syndrome (PDNS), proliferative and necrotizing pneumonia (PNP), reproductive failure, and enteritis (Landrau-Giovannetti et al., 2020). In aquatic systems, European catfish circovirus (EcatfishCV) has been associated with mass mortality events in Hungary (Lőrincz et al., 2012). Although the pathogenicity of circoviruses in snakes remains unclear, the potential for subclinical or immunosuppressive effects warrants further investigation.

*Gloydius strauchi*, commonly referred to as the alpine pit viper, is a high-altitude species within the family *Viperidae*, endemic to the plateau regions of central and western China, including Sichuan, Yunnan, Xizang, Shaanxi, Gansu, Qinghai, and Ningxia. This species usually occupies rocky habitats and primarily preys on rodents. Despite its broad distribution and ecological specialization, no viral pathogens have been formally documented in the alpine pit viper.

This study identified and characterized the complete genome of a novel *Circovirus* isolated from the oral cavity of wild *G. strauchi*. Phylogenetic and recombination analyses demonstrated that the virus was closely related to *Circovirus* strains of mammalian and avian origin, indicating a potential cross-species transmission cycle within the *Gloydius* lineage.

## 2 Materials and methods

### 2.1 Sample collection

In June 2023, a wild Asian pit viper was captured in Shimian County, Sichuan Province, China. Oral samples were collected following previously described procedures (Liu et al., 2023). Swabs were placed in RNase-free tubes and immediately transported on dry ice to the laboratory. The snake was subsequently released back into the wild upon sample collection.

### 2.2 Detection of *Circovirus* in oral samples and amplification of complete genome sequences by polymerase chain reaction (PCR)

DNA was extracted from oral swabs using a commercial kit and screened for *Circovirus* using a primer pair targeting the Rep protein gene (Cir-F: GTTTACCTTGAGATTGGAGAG; Cir-R: TTTCCACGGGGTTTCCAGTATT). To amplify the complete genome, additional primers were used: Cir-F1: AGAGGTCAGACCTGAAGGAA (position 471–490), Cir-R1: CCTGTTTTTCAGATGCCTCATT (position 1761–1782), Cir-F2: GTGAAGGTGAGCCTATCAGAGT (position 1541–1562), and Cir-R2: CAGTCCGTTCAGAACAAAACTT (position 671–692). PCR amplification was performed under the following cycling parameters: initial denaturation at 94 °C for 5 min, followed by 35 cycles at 94 °C for 30 s, 62 °C for 30 s, and 72 °C for 2 min, followed by a final extension at 72 °C for 10 min (Hu et al., 2015). PCR products were cloned into the pMD18-T vector (TaKaRa) prior to sequencing. Three independent clones were sequenced using the ABI_3500 platform with universal primers (M13F: AGGGTTTTCCCAGTCACG; M13R: CAGGAAACAGCTATGAC) to confirm sequence integrity.

### 2.3 Phylogenetic and genetic analyses

Pairwise nucleotide sequence similarity was determined using the Needleman-Wunsch global alignment algorithm^1^. Phylogenetic trees were constructed based on the complete genome, Rep protein, and Cap protein sequences using MEGA (v7.0) and the maximum-likelihood (ML) approach with the GTR + G + I, LG + G, and WAG + G + F models, respectively. Bootstrap support was estimated from 1,000 replicates. Simplot v3.5.1 was used to compare GsCV1 nucleotide sequences with the reference *Circovirus* strains. The GsCV1 sequence has been submitted to GenBank database under the accession number PX310168.

## 3 Results

### 3.1 Genome architecture and genetic features of GsCV1

**FIGURE 1:**
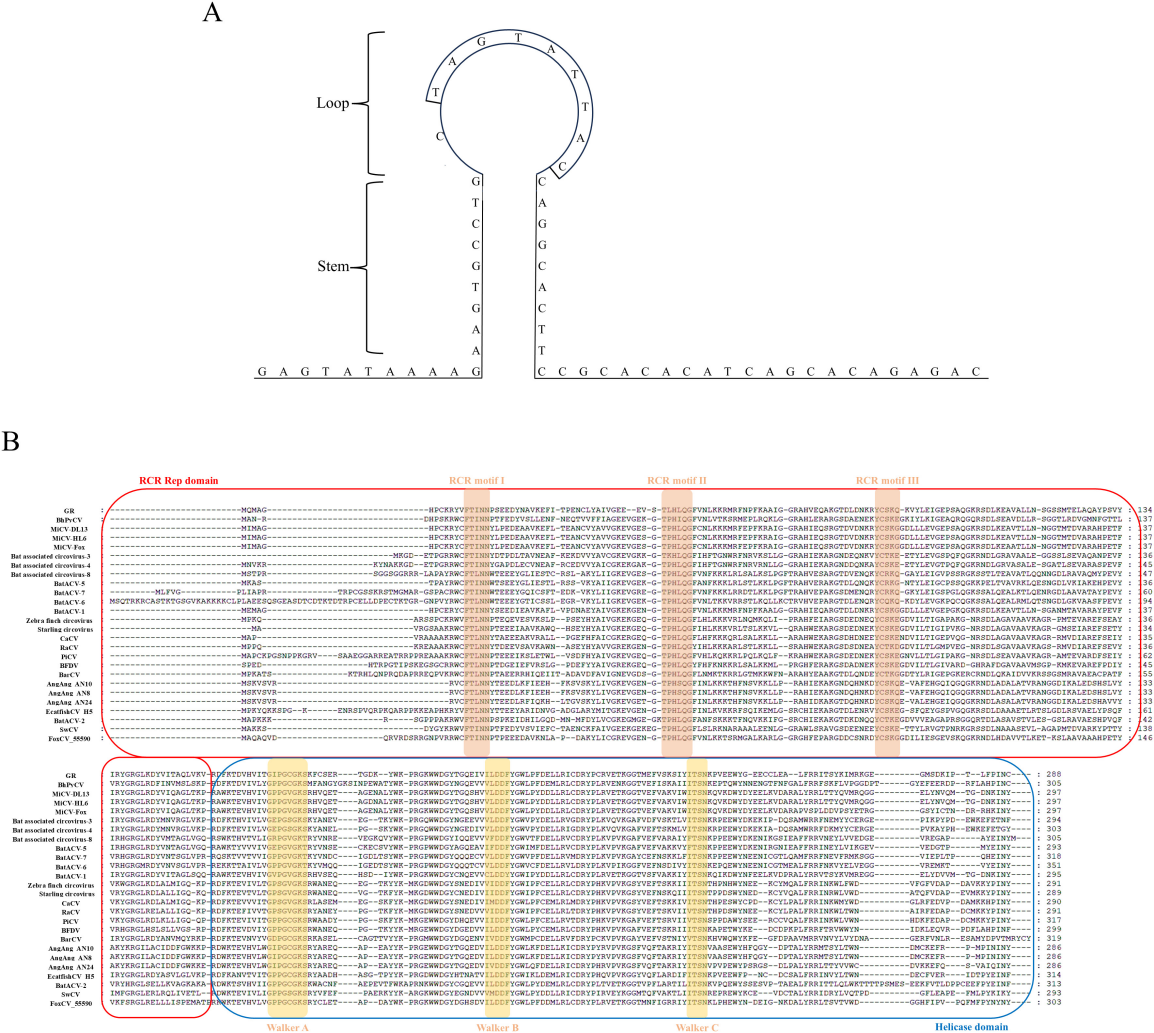
Predicted stem-loop structure of GsCV1 **(A)** and multiple sequence alignment of Rep proteins from representative circoviruses **(B)**. Conserved rolling-circle replication (RCR) motifs I, II, and III are indicated in boxes, along with Walker A, B, and C motifs associated with dNTP binding. Putative RCR initiator and helicase domains are annotated.

The complete genome of strain GsCV1 comprised 1,811 nucleotides and contained two major open reading frames (ORFs): an 867-nucleotide ORF encoding the Rep protein on the virion strand and a 753-nucleotide ORF encoding the Cap protein on the complementary strand. A conserved 9-bp non-anucleotide motif (TAGTATTAC) was identified at the apex of the predicted stem-loop structure (Figure 1A). Three conserved rolling circle replication (RCR) motifs, along with an SH3-like helicase domain, were detected within the Rep protein sequence (Figure 1B). Notably, GsCV1 exhibited an overlapping region between the Rep and Cap coding regions, similar to that observed in WigFec circovirus 1.

**FIGURE 2:**
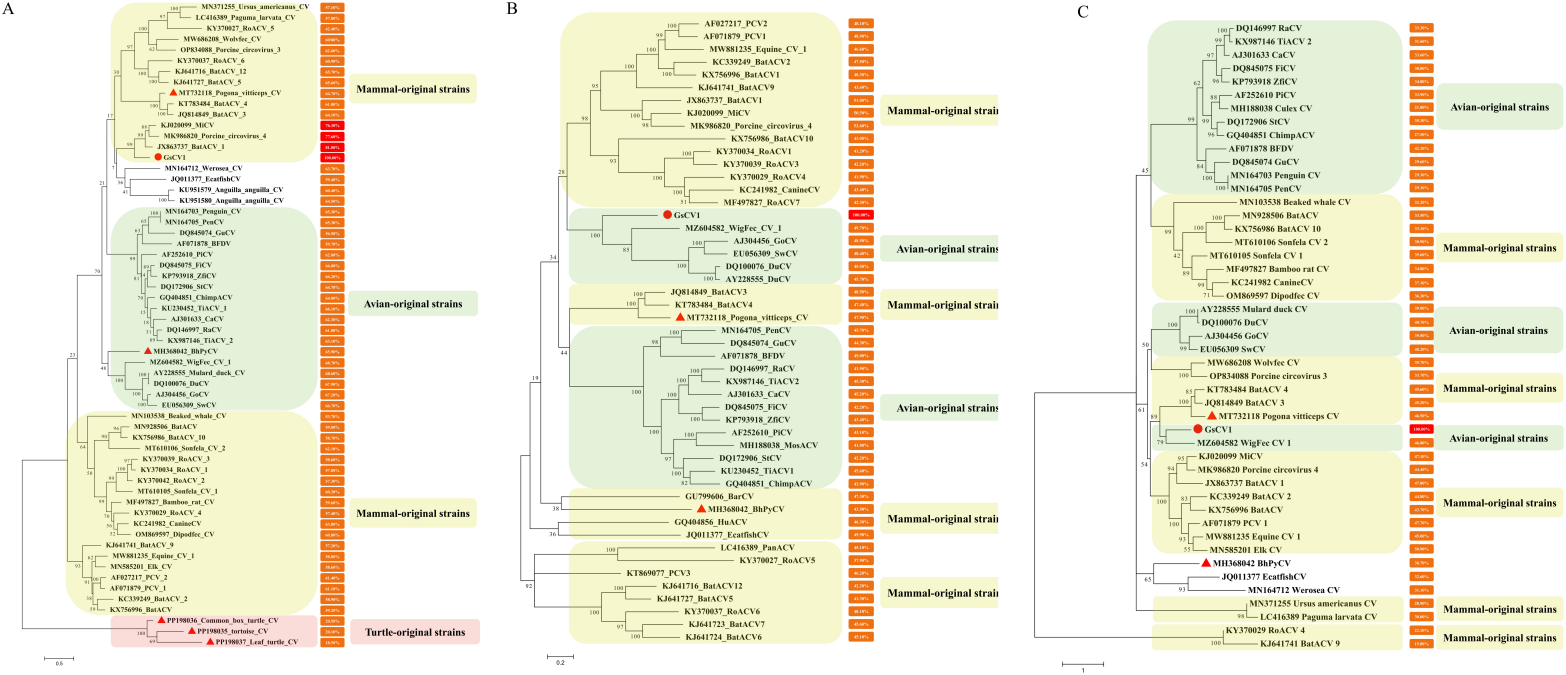
Phylogenetic trees constructed using the maximum-likelihood method based on complete genome nucleotide sequences **(A)**, Rep protein amino acid sequences **(B)**, and Cap protein amino acid sequences **(C)**. Percentage pairwise identities relative to GsCV1 are shown next to each taxon name, along with corresponding GenBank accession numbers. “●” represents the strain identified in the present study, “◆” represents the reptiles’ strains.

Pairwise sequence comparisons revealed that the GsCV1 Rep protein shares over 70% amino acid identity with bat-associated circovirus 1 (BatACV1), mink circovirus (MiCV), and porcine circovirus 4 (PCV4) (Figure 2B), while its nucleotide sequence and Cap protein exhibit less than 60% identity with all recognized *Circovirus* species (Figures 2A, C). These divergence patterns suggest that GsCV1 may represent a genetically distinct lineage with potentially novel functional attributes requiring further investigation.

### 3.2 Phylogenetic relationships and recombinant analyses of GsCV1

Phylogenetic reconstruction based on the Rep protein positioned GsCV1 within a distinct cluster alongside mammalian-origin circoviruses, including BatACV1, MiCV, and PCV4 (Figure 2B). In contrast, analyses based on the complete genome and Cap protein sequence placed GsCV1 within a clade of avian-derived circoviruses, indicating topological incongruence across genomic regions (Figures 2A, C). Similar discordant clustering patterns were observed in other reptile-derived circoviruses: snake-origin BhPyCV grouped with avian strains, lizard-associated *Pogona vitticeps* circovirus (PvCV) aligned with mammalian strains, and turtle-origin circoviruses formed a distinct monophyletic lineage.

**FIGURE 3:**
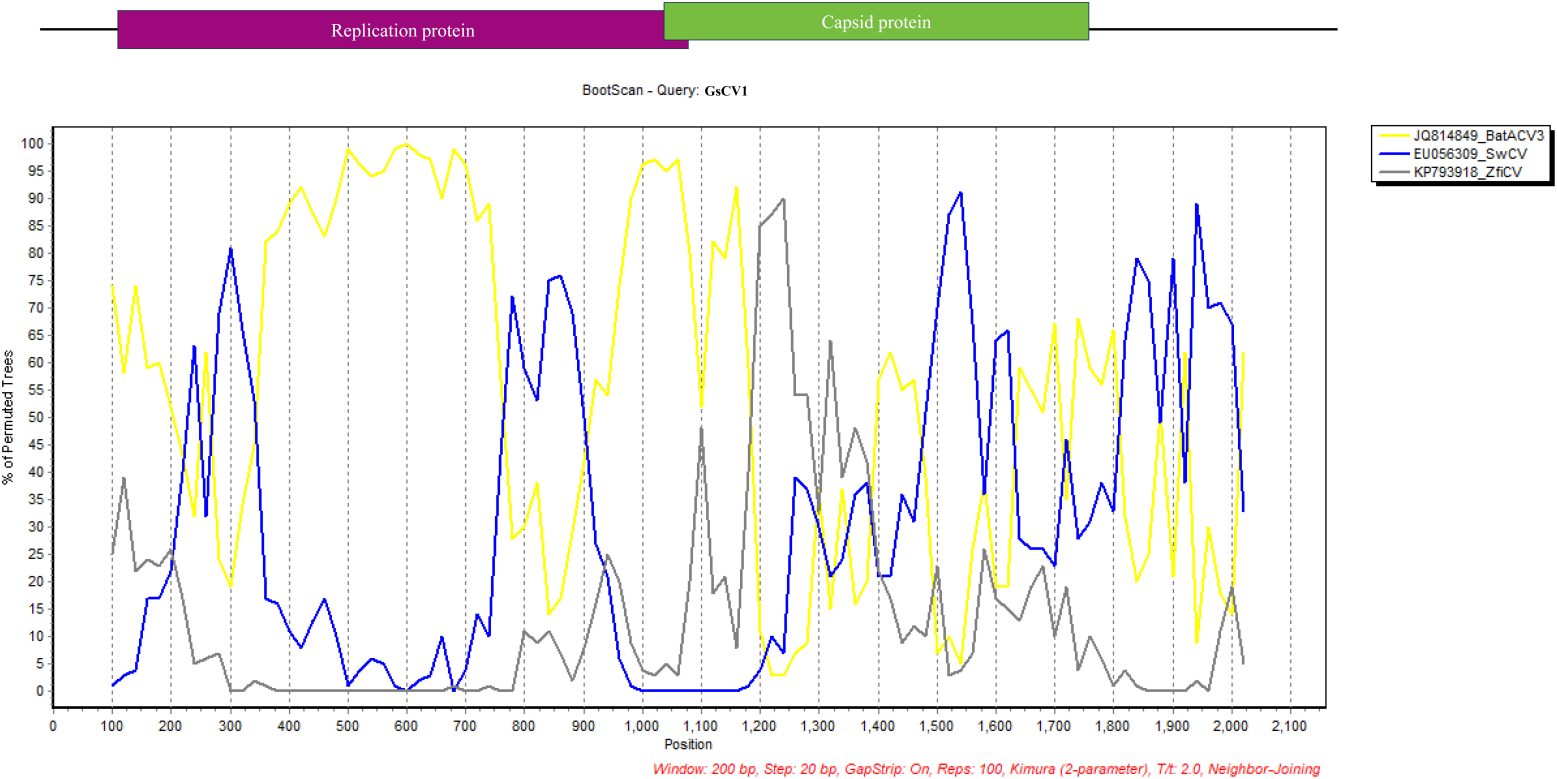
Recombination analysis of the GsCV1 strain. Crossover region in the GsCV1 genome was detected using Simplot v3.5.1. Y-axis represents the percentage of permuted trees, calculated with a sliding window of 200 nucleotides and step size of 20 nucleotides. Analyses were conducted using the Kimura 2-parameter model, a transition/transversion (Ts/Tv) ratio of 2.0, neighbor-joining tree model, and 1000 bootstrap replicates. Reference strains: BatACV3 (JQ814849), SwCV (EU056309), and ZfiCV (KP793918).

Recombination analysis indicated that GsCV1 likely originated through interspecies recombination involving BatACV3, swan circovirus (SwCV), and zebra finch circovirus (ZfiCV), highlighting the potential role of host-driven recombination in shaping circovirus evolution (Figure 3).

## 4 Discussion

Members of the family *Circoviridae* are known to infect a broad range of mammalian and avian hosts, including economically and ecologically significant pathogens such as PCV2 and beak and feather disease virus (BFDV) (Heath et al., 2004; Ma et al., 2007). Phylogenetic analyses of circoviruses derived from reptiles have revealed dispersed affiliations with both mammalian and avian lineages. For example, PvCV shows evolutionary proximity to bat-associated strains, whereas black-headed python circovirus (BhPyCV) clusters with avian-origin strains. In contrast, turtle-derived circoviruses—such as those from common box turtles, tortoises, and leaf turtles—form a distinct clade, suggesting a more lineage-specific evolutionary trajectory. In the present study, the Rep protein of GsCV1 exhibited closest similarity to bat- and mink-associated circoviruses, while its complete nucleotide sequence and Cap protein were more closely related to avian strains. These findings underscore the genetic heterogeneity among reptile-associated circoviruses and suggest that multiple ancestral lineages may be co-circulating within different reptilian taxa. However, due to the limited number of available reptile-derived *Circovirus* genomes, current evidence remains insufficient to resolve deep evolutionary relationships between reptile-associated and broader wildlife circoviruses.

Previous studies have identified recombination as a key mechanism driving *Circovirus* diversification and host adaptation (Piewbang et al., 2018). In PCV2, intra- and inter-genotypic recombination events have been documented, particularly in the context of interactions between wild boars and domestic pigs (Cadar et al., 2012; Cai et al., 2012). The present study reports, for the first time, a mammalian-avian recombinant *Circovirus* strain, suggesting that cross-species recombination may be more widespread than previously recognized. If the *Circovirus* infects in the new host, local adaption is the first barrier. The mechanism for transmission dynamic and host-virus interaction is ambiguous. In addition, mixed infections likely facilitate such events, with recombination potentially influenced by factors such as origin of replication motifs, local sequence homology, secondary structural features, and discontinuities in replication or transcription dynamics. Further investigation is warranted to elucidate the molecular mechanisms of recombination in circoviruses, particularly in the context of host switching between avian and mammalian reservoirs.

Previous studies have shown that PvCV clusters with bat-associated strains (Chang et al., 2020), raising the possibility of interspecies transmission facilitated by the high mobility of chiropteran hosts. Our previous study identified a *Circovirus* fragment in *G. angusticeps* from the eastern part of the Qinghai-Tibet Plateau, representing the first *Circovirus* detected in Chinese snakes (Liu et al., 2023). Notably, while GsCV1 showed close genetic similarity to this strain, it also clustered with a bat-associated *Circovirus* detected in Myanmar in 2008 and with *Anas diazi Circovirus* detected in the United States in 2021, as well as with strains isolated from both domestic animals and wildlife. These findings raise the possibility that GsCV1 may have disseminated into western Sichuan via bat migration, unknown intermediate hosts, or predator-prey transmission pathways involving snakes consuming bats or preying on other infected animals such as rodents. Although zoonotic RNA viruses have received greater attention in surveillance and epidemiological studies, DNA viruses—such as circoviruses—remain underexplored in this context. A deeper understanding of the ecological links, host interactions, and geographical drivers of *Circovirus* emergence will be critical to identifying latent reservoirs and assessing the risk of future cross-species transmission events.

## 5 Conclusion

A recombinant *Circovirus*, GsCV1, was identified in *G. strauchi*, a high-altitude pit viper species endemic to western Sichuan Province, China. Phylogenetic analysis indicated that GsCV1 exhibits genetic affinities with both mammalian- and avian-associated circoviruses. These findings suggest that snake-associated circoviruses may have originated from multiple ancestral lineages and persist through latent transmission cycles involving mammalian and avian hosts.

## Data Availability

The data presented in this study are deposited in the online repository. The names of the repository/repositories and accession number(s) can be found here: NCBI, accession number PX310168.

## Footnotes

1
https://www.ebi.ac.uk/jdispatcher/psa
